# Trends in Colistin Resistance and Multidrug-Resistant Phenotypes Among Gram-Negative Bacilli: A Retrospective Analysis

**DOI:** 10.3390/molecules30142950

**Published:** 2025-07-12

**Authors:** Madalina Alexandra Vlad, Maria Dan, Andreea Nicoleta Catana, Sebastian Dumitriu, Cristina Gabriela Tuchilus

**Affiliations:** 1 Department of Microbiology, Faculty of Medicine, “Grigore T. Popa” University of Medicine and Pharmacy, 16 Universitatii Street, 700115 Iași, Romania; cristina.tuchilus@umfiasi.ro; 2 Medical Analysis Laboratory, “St. Spiridon” County Clinical Emergency Hospital, 700111 Iași, Romania; m2maria2000@yahoo.com (M.D.); sebastiandumitriu239@gmail.com (S.D.); 3 Clinical Medical Department, “St. Spiridon” County Clinical Emergency Hospital, 700111 Iași, Romania; catana.andreea87@gmail.com

**Keywords:** colistin resistance, gram-negative bacilli, MDR, antimicrobial resistance, *Klebsiella* spp., *E. coli*, *Enterobacter* spp., *Citrobacter* spp., *Pseudomonas* spp., *Acinetobacter* spp.

## Abstract

Colistin has re-emerged as a last-resort antibiotic for treating infections caused by multidrug-resistant (MDR) Gram-negative bacilli (GNB). However, increasing resistance threatens its efficacy. This study aimed to evaluate colistin resistance trends among clinical isolates of Gram-negative bacilli isolated over a five-year period at a large Emergency Hospital in North-Eastern Romania. A total of 23,143 GNB strains were isolated during the study period, including 14,531 *Enterobacterales* and 8294 non-fermenting Gram-negative bacilli. The percentage of colistin-resistant strains among those analyzed was 3.98%. Species-specific analysis focused on *Klebsiella* spp., *Escherichia coli*, *Enterobacter* spp., *Citrobacter* spp., *Pseudomonas* spp., and *Acinetobacter* spp.* Klebsiella* spp. exhibited the highest prevalence of colistin resistance, accounting for over 80% of all colistin-resistant strains, with annual resistance rates fluctuating between 12.97% and 21.64%. Colistin resistance among *E. coli* was low (0.18–1.25%). *Citrobacter* spp. showed no resistance in the last three years of the study, and *Enterobacter* spp. maintained relatively stable resistance (3–5%). Resistance in *Pseudomonas* spp. remained below 1%, while *Acinetobacter* spp. showed a resistance rate of 5.43%. Several distinct resistance phenotypes were identified among *Klebsiella* spp., *Pseudomonas* spp., and *Acinetobacter* spp. strains, reflecting both endemic and sporadic circulation patterns. The study highlights a persistent presence of colistin resistance, especially in *Klebsiella* spp., underlining the importance of ongoing surveillance. Despite low resistance in other species, the emergence of resistant strains underscores the need for robust antimicrobial stewardship and infection control policies.

## 1. Introduction

Antibiotic resistance represents one of the greatest challenges of modern medicine. Among these, resistance to colistin in Gram-negative bacilli has emerged as a particularly alarming issue. The resurgence in the use of colistin has been necessitated by the increasing prevalence of multidrug-resistant Gram-negative pathogens. In recent years, the rate of antibiotic resistance has grown significantly, contributing to therapeutic failure in the treatment of severe infections.

The swift dissemination of multidrug-resistant (MDR) bacteria—including carbapenem-resistant *Enterobacterales*, *Acinetobacter baumannii,* and *Pseudomonas aeruginosa* has emerged as a significant public health concern, particularly in countries where the spread of carbapenem-resistant organisms has become endemic [1,2]. Carbapenemase-producing *Enterobacterales* (CRE) represent a major public health concern, as these organisms either produce carbapenemases—enzymes belonging to the β-lactamase class that hydrolyze carbapenem antibiotics—or exhibit acquired resistance to carbapenems despite lacking intrinsic mechanisms [3,4]. Given the role of carbapenems as last-line agents for multidrug-resistant infections, the increasing prevalence of CRE has led to a greater reliance on colistin as salvage therapy [5]. This shift has intensified selective pressure, thereby contributing to the emergence and dissemination of colistin-resistant strains, further limiting therapeutic options and complicating infection control efforts [3].

Although carbapenem resistance in *Enterobacterales* can arise through various mechanisms, the most prevalent is the production of carbapenemase enzymes, which efficiently hydrolyze carbapenem antibiotics [1,6,7]. These carbapenemase-producing strains utilize diverse molecular strategies to inactivate antimicrobial agents [4]. In addition to enzymatic degradation, other notable resistance mechanisms include the overexpression of efflux pumps and alterations in outer membrane porins, which reduce antibiotic permeability and hinder the drugs from reaching their intracellular targets [3].

The resistance mechanisms exhibited by MDR or XDR (extensively drug-resistant) *Acinetobacter baumannii* complex—recognized as a critical priority pathogen by the World Health Organization [8]—commonly involve reduced outer membrane permeability due to porin loss, constitutive overexpression of efflux pump systems, and the production of various β-lactamases [9].

*Pseudomonas aeruginosa* exhibits multiple resistance mechanisms, including carbapenemase production (notably VIM and OXA types), overexpression of AmpC β-lactamases, and increased activity of efflux pumps, which collectively contribute to its resistance to imipenem and other β-lactam antibiotics [10].

The extensive application of colistin in clinical practice and agriculture has facilitated the global spread of the mobile colistin resistance gene *mcr*-1 [11]. Colistin’s bactericidal effect is mediated by electrostatic interactions between its positively charged residues and the negatively charged lipid A of lipopolysaccharide, resulting in disruption of the bacterial outer membrane [11].

Colistin, a pentacationic cyclic lipodecapeptide mainly consisting of colistin A and B, belongs to the polymyxin class of antibiotics. It is effective against most Gram-negative bacteria, except for those from the genera *Proteus*, *Providencia*, *Morganella*, *Serratia*, *Edwardsiella,* and *Burkholderia*, which exhibit intrinsic resistance. Its positive charge facilitates binding to the negatively charged lipopolysaccharide (LPS) in the outer membrane, displacing divalent cations and disrupting membrane stability, ultimately leading to bacterial cell death [12].

For several decades (1960–1990), colistin resistance was rarely observed. However, recent years have seen a notable rise in reports of the emergence, isolation, and dissemination of colistin-resistant *Klebsiella pneumoniae* (CRKP) in clinical settings across various countries in the Americas, Asia, and Europe [13,14,15,16,17,18]. The increased detection of CRKP has been largely attributed to the renewed clinical use of colistin and polymyxin B [19,20,21,22] as well as the multiclonal transmission of resistant strains among hospitalized patients [16,23]. While the development of CRKP is often linked to prior colistin exposure [21], cases have also been documented in patients with no history of colistin therapy [24,25].

Of particular concern is the rapid emergence of colistin-resistant *Escherichia coli*, which has gained global attention as a growing public health threat [26].

Several recent studies have reported that *Enterobacter* strains harbor carbapenemase genes in addition to exhibiting resistance to colistin [27,28,29].

*Citrobacter* spp. are Gram-negative bacteria commonly present in the environment and in the intestinal tracts of humans and animals. While they are typically susceptible to third-generation cephalosporins, carbapenems, and colistin, an increasing number of studies have reported the presence of antibiotic resistance genes in these organisms. This raises concerns that *Citrobacter* spp. may serve as a potential reservoir for the dissemination of antimicrobial resistance genes [30].

Colistin remains one of the few effective treatment options for life-threatening infections caused by multidrug- and extensively drug-resistant *Acinetobacter baumannii* and *Pseudomonas aeruginosa* [31]. Furthermore, resistance to colistin is relatively uncommon and is primarily mediated by chromosomally encoded mechanisms, which limits its potential for horizontal gene transfer [32].

This study aimed to assess colistin resistance rates over the 2019–2023 period in the most frequently isolated Gram-negative bacilli strains—*Klebsiella* spp., *Escherichia coli*, *Enterobacter* spp., *Citrobacter* spp., *Pseudomonas* spp., and *Acinetobacter* spp.—in order to better understand their resistance profiles in hospitalized patients, and we also investigated the prevalence and distribution of multidrug-resistant (MDR) phenotypes among these isolates, providing a comprehensive overview of resistance patterns.

## 2. Results

### 2.1. Isolation and Identification

The results were analyzed by bacterial species, tracking the annual evolution of colistin resistance rates in comparison with other antimicrobial agents.

Between 2019 and 2023, a total of 23,143 Gram-negative bacilli (GNB) strains were isolated at “Sf. Spiridon” County Clinical Emergency Hospital in Iași, Romania. Of these, 14,849 were identified as *Enterobacterales,* and 8294 were non-fermenting Gram-negative bacilli. Out of the total, 4795 strains were classified as multidrug-resistant Gram-negative bacilli (MDR GNB).

**Figure 1:**
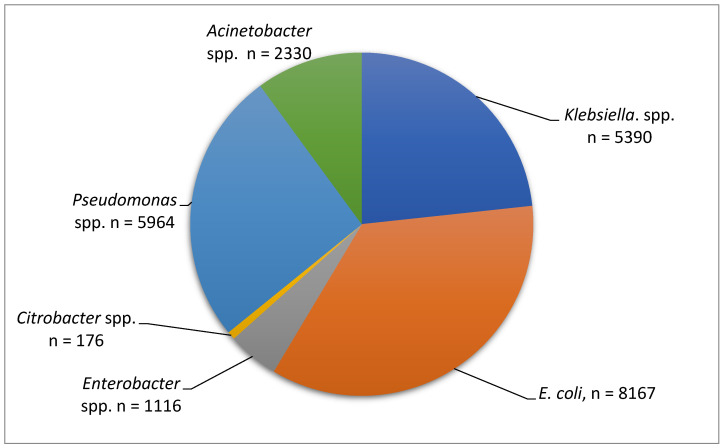
Number of strains analyzed during the period 2019–2023.

This study illustrates the distribution of Gram-negative bacilli isolates analyzed between 2019 and 2023, with *Escherichia coli* (8167 isolates) being the most frequently identified organism, followed by *Pseudomonas* spp. (5964 isolates) and *Klebsiella* spp. (5390 isolates), all of which are major contributors to hospital-acquired infections. *Acinetobacter* spp. accounted for 2330 isolates and is particularly notable for its association with multidrug resistance, especially in intensive care settings. *Enterobacter* spp. and *Citrobacter* spp. were less frequently isolated, with 1116 and 176 isolates, respectively, though *Citrobacter* spp. remains clinically relevant in the context of resistant strains. Overall, the data show that *E. coli*, *Pseudomonas,* and *Klebsiella* species represent the majority of isolates, reflecting common patterns observed in clinical practice (Figure 1).

**Figure 2:**
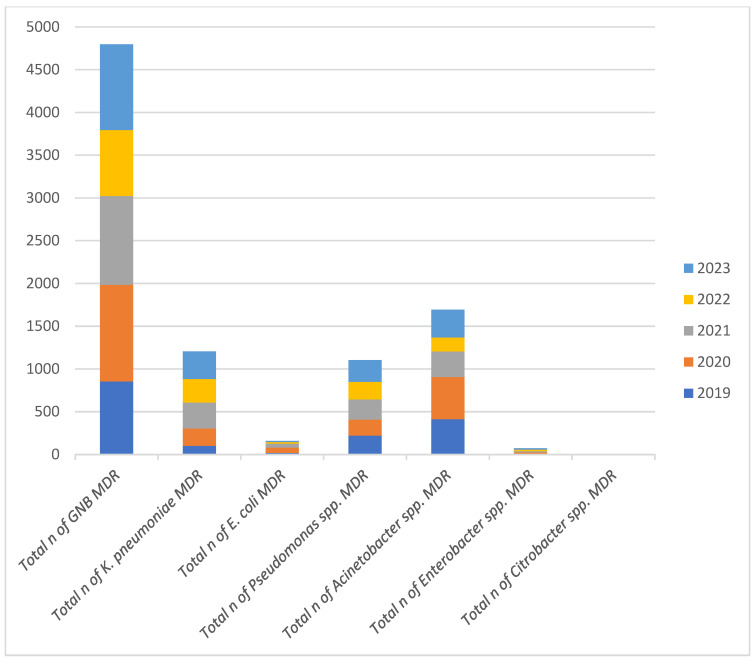
Annual Distribution of Multidrug-Resistant Gram-Negative Bacilli Isolates (2019–2023). Note: *n* = number of strains.

Over the five-year period, the total number of MDR GNB isolates peaked in 2019 and reached its lowest in 2022. Among the individual species, *Acinetobacter* spp. consistently accounted for a substantial proportion of MDR isolates. In 2023, *Acinetobacter* spp. represented the highest proportion of MDR isolates relative to the total MDR count, surpassing *Klebsiella*, *E. coli*, and *Pseudomonas* spp. These trends suggest that while overall MDR rates have fluctuated, *Acinetobacter* spp. remains a dominant and persistent MDR pathogen, warranting continuous surveillance and targeted infection control strategies. At the opposite end of the spectrum, *Citrobacter* spp. exhibited extremely low MDR rates throughout the study period, with certain years registering no MDR isolates at all (Figure 2).

**Figure 3:**
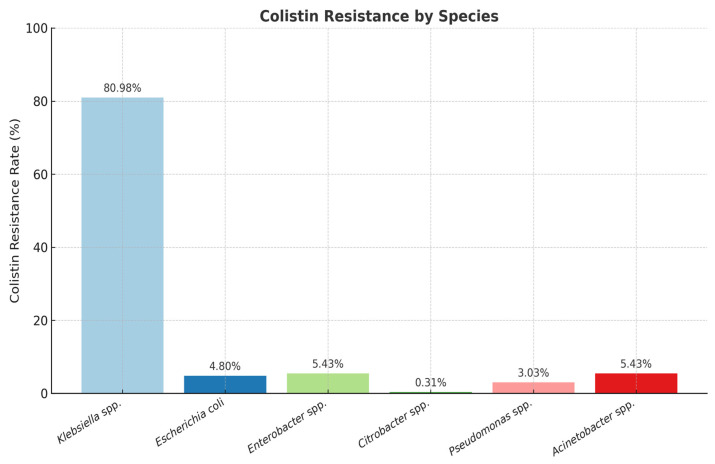
Distribution of colistin-resistant Gram-negative strains. Note: The number of isolates per species differs and reflects the clinical frequency of isolation during the study period.

The percentage of colistin-resistant strains among those analyzed was 3.98%. Among the *Enterobacterales* group, the highest number of colistin-resistant isolates were identified as *Klebsiella* spp. (80.98%), but resistance to colistin was also detected in *Escherichia coli* (4.80%), *Enterobacter* spp. (5.43%), and *Citrobacter* spp. (0.31%) (Figure 3).

While species-specific isolate numbers vary, the data reflect the actual clinical distribution of multidrug-resistant Gram-negative bacilli during the study period in our hospital.

Colistin resistance among the analyzed strains varied but showed a slightly increasing annual trend over the study period across all included species.

The overwhelming majority of resistant isolates were identified as *Klebsiella* spp. (80.98%), highlighting their significant role in colistin resistance among clinical pathogens.

Other species showed considerably lower proportions, with *Enterobacter* spp. and *Acinetobacter* spp. each representing 5.43% of resistant strains.

*Escherichia coli* represented 4.80% of the colistin-resistant isolates, while *Pseudomonas* spp. and *Citrobacter* spp. accounted for 3.03% and 0.31%, respectively. These findings suggest that, although colistin resistance is distributed among various Gram-negative species, *Klebsiella* spp. remain the most significant source of resistance in our study (Figure 3).

### 2.2. Klebsiella *spp.*

*Klebsiella* spp. isolates exhibited a rising trend in resistance to ciprofloxacin and cephalosporins during the five-year analysis. Although a gradual increase in colistin resistance among *Klebsiella* spp. was observed over the study period, this trend did not reach statistical significance (*p* = 0.894), suggesting that the variation may be due to random fluctuation rather than a consistent upward pattern. Colistin-resistant *Klebsiella* spp. strains were additionally resistant to other classes of antibiotics, including carbapenems, leaving clinicians with very few therapeutic options available (Table 1).

To assess the association between year of isolation and resistance rate for each antibiotic, *p*-values were calculated using the Pearson Chi-square test. A *p*-value < 0.05 was considered statistically significant.

Among all tested antibiotics, a statistically significant difference in resistance rates over the five-year period was observed only for meropenem (*p* < 0.05), indicating temporal variation. For the other antibiotics, the differences were not statistically significant (*p* > 0.05) (Table 1).

The annual assessment of colistin resistance in *Klebsiella* spp. isolates over the 2019–2023 period revealed the highest resistance rate in 2020 (21.64%), followed by a significant decline in 2021 to 12.97%. While the rates remained relatively stable in 2019 (12.99%) and 2021, a gradual increase was noted again in 2022 (14.06%) and 2023 (15.82%). Despite year-to-year fluctuations, the overall trend indicates that colistin resistance in *Klebsiella* spp. remained consistently present at concerning levels over the five-year period. (Table 1). 

**Figure 4:**
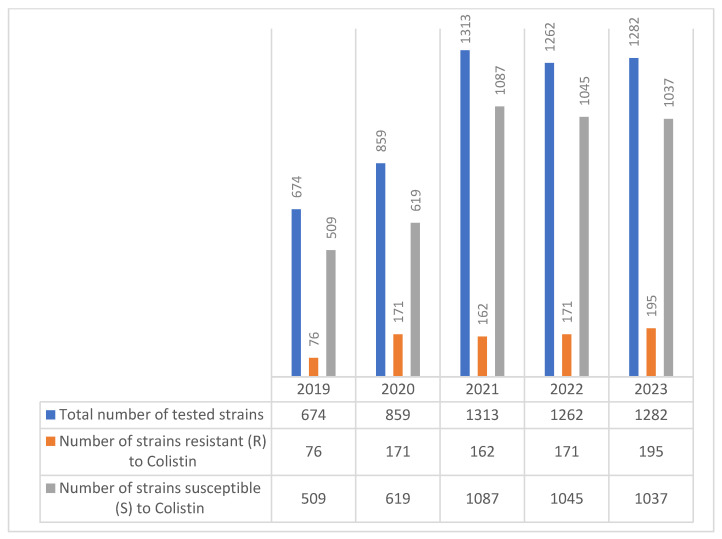
Evolution of colistin resistance in *Klebsiella* spp. strains isolated during the 2019–2023 period.

During the 2019–2023 study period, the number of *Klebsiella* spp. strains tested for colistin susceptibility increased steadily, ranging from 674 in 2019 to 1282 in 2023. Among these, the number of colistin-resistant strains also showed a gradual rise, from 76 in 2019 to 195 in 2023, reflecting a concerning upward trend in resistance (Figure 4).

Throughout the study, various MDR *Klebsiella* spp. phenotypes were repeatedly isolated, suggesting their ongoing endemic presence within the hospital environment (Table 2). Descriptive phenotypic resistance profiles were presented only for *Klebsiella* spp., *Pseudomonas* spp., and *Acinetobacter* spp., as these were the most frequently isolated species and posed the greatest therapeutic challenges, while less frequent isolates such as *E. coli*, *Enterobacter* spp., and *Citrobacter* spp. did not display complex resistance patterns warranting detailed phenotypic analysis. These strains were repeatedly isolated from multiple patients and from a broad spectrum of pathological specimens, including blood, respiratory secretions, urine, and wound exudates. Their recurrent detection across different clinical contexts suggests persistent transmission pathways and adaptation to selective antibiotic pressure within the healthcare setting. The phenotypes displayed limited susceptibility, with some strains remaining susceptible only to amikacin and trimethoprim-sulfamethoxazole while exhibiting intermediate susceptibility to imipenem.

### 2.3. Escherichia coli

Over the five-year period, a total of 8167 *E. coli* strains were tested for colistin susceptibility. While the number of isolates tested increased steadily each year—from 1191 in 2019 to 2281 in 2023—the number of colistin-resistant strains remained low overall, ranging from just 3 cases in 2021 to a peak of 15 in 2019.

**Figure 5:**
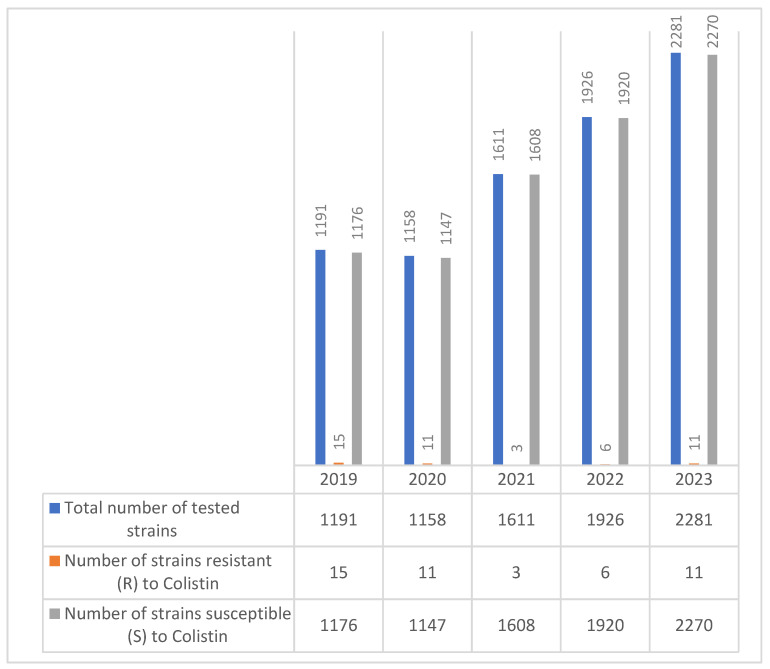
Evolution of colistin resistance in *E. coli* strains isolated during the 2019–2023 period.

Despite the low absolute numbers of resistant strains, the data demonstrate sporadic detection of resistance across all years, with a slight resurgence in 2023 (11 resistant strains) (Figure 5).

The colistin-resistant *E. coli* strains were characterized by susceptibility to other antibiotic classes, indicating that they were not multidrug-resistant (MDR). This pattern suggests a possible plasmid-mediated mechanism of colistin resistance.

In *E. coli* isolates, a statistically significant decreasing trend in resistance over the five-year period was observed for cefotaxime (*p* = 0.048), ceftriaxone (*p* = 0.024), and cefepime (*p* = 0.046), indicating a potential shift in susceptibility patterns to third- and fourth-generation cephalosporins.

For the remaining antibiotics, resistance rates remained relatively stable, with *p*- above the 0.05 threshold (Table 3).

Amoxicillin–clavulanic acid showed variable resistance, peaking in 2020 (39.6%) and decreasing to around 29% by 2023. This fluctuation may reflect shifts in prescription patterns or local strain dynamics (Table 3).

Third-generation cephalosporins (cefotaxime, ceftazidime, and ceftriaxone) showed a clear declining trend in resistance: cefotaxime dropped from 26% to 16.5%; ceftriaxone dropped from 33.3% to 16.75%. This suggests improved susceptibility, potentially due to reduced selection pressure or better control of ESBL-producing strains. Cefepime resistance also declined significantly, reaching 12.5% in 2023 from 19% in 2019 (Table 3).

Imipenem, meropenem, and ertapenem all maintained very low resistance rates, although slight increases were noted in some years (e.g., imipenem reaching 2.89% in 2023). These consistently low values support carbapenems’ continued effectiveness against *E. coli*, although caution is still warranted due to the potential emergence of carbapenemase-producing strains.

Ciprofloxacin resistance remained moderately high, with a slight decrease from 30.3% in 2020 to 21.7% in 2023. This reflects ongoing selection pressure from widespread fluoroquinolone use.

Colistin resistance remained very low across all years, decreasing from 1.25% in 2019 to 0.48% in 2023, supporting its role as a last-resort treatment.

Trimethoprim–sulfamethoxazole showed consistently high resistance, ranging from approximately 25% to 36%, limiting its utility for empirical treatment.

Nitrofurantoin, commonly used for urinary tract infections, showed very low resistance rates throughout the study period (2–3.75%), confirming its continued effectiveness against *E. coli* (Table 3).

These findings suggest that colistin resistance in *E. coli* remains relatively rare but must be closely monitored, especially given the observed fluctuation and potential for increased resistance in clinical environments.

### 2.4. Enterobacter *spp.*

**Figure 6:**
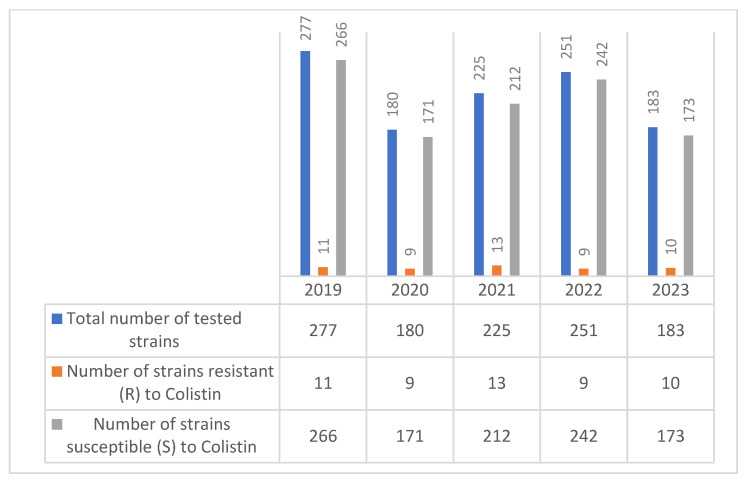
Evolution of colistin resistance in *Enterobacter* spp. strains isolated during the 2019–2023 period.

Regarding the evolution of colistin resistance in *Enterobacter* spp. strains over the five-year period (2019–2023), a total of 1116 isolates were tested, with annual strain sizes ranging from 180 to 277. Colistin resistance was reported in each year, with the number of resistant strains varying between 9 and 13 annually (Figure 6).

The number of colistin-resistant strains remained relatively stable—peaking at 13 in 2021—and did not show a clear increasing or decreasing trend. Most *Enterobacter* spp. isolates remained susceptible to colistin.

This data suggests a persistent low-level presence of colistin resistance in *Enterobacter* spp.

Among the antibiotics tested, only ertapenem showed a statistically significant variation in resistance rates over the study period (*p* = 0.011), while for the others, the *p*-values were above the conventional threshold of 0.05, indicating no significant temporal trend (Table 4).

### 2.5. Citrobacter *spp.*

**Figure 7:**
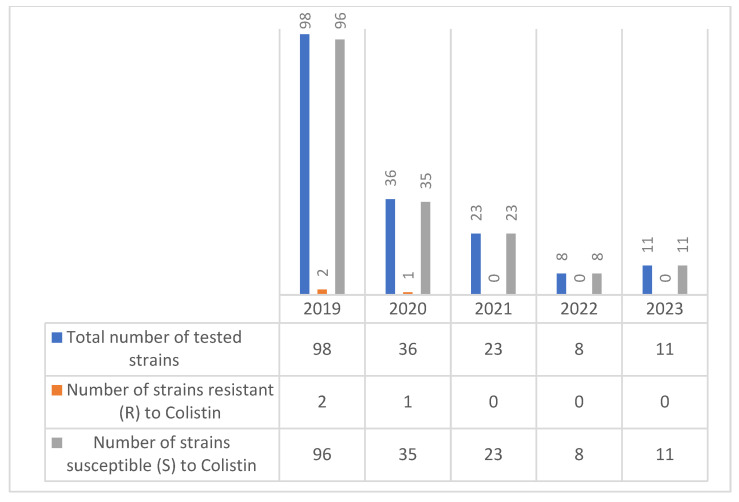
Evolution of colistin resistance in *Citrobacter* spp. strains isolated during the period 2019–2023.

According to our study, the number of colistin-resistant *Citrobacter* spp. strains was low in 2019 and 2020 (2 strains and 1 strain, respectively), and starting from 2021, no colistin-resistant *Citrobacter* spp. strains were detected (Figure 7).

In *Citrobacter* spp., statistically significant changes in resistance rates over the 2019–2023 period were observed for ceftazidime (*p* = 0.031) and cefotaxime (*p* = 0.042), suggesting potential shifts in susceptibility patterns for these third-generation cephalosporins. For all other antibiotics, the *p*-values exceeded 0.05, indicating no significant temporal variation (Table 5).

Resistance to carbapenems was very low throughout the period, and by 2022 and 2023, no resistance was detected for imipenem, meropenem, and ertapenem. This suggests carbapenems remain largely effective against *Citrobacter* spp. in this clinical context.

The slight elevation in ertapenem resistance in 2021 (8%) could indicate a temporary emergence of a resistant subpopulation.

Colistin resistance was minimal, with low rates (2–2.7%) reported in 2019 and 2020. No resistance was detected in the last three years (2021–2023), indicating stable susceptibility and minimal selective pressure from colistin use among *Citrobacter* spp. isolates. Resistance levels gradually increased for cefotaxime and ceftazidime, with the most pronounced rise seen in ceftriaxone, reaching 50% in 2023.

Cefepime resistance, while initially low (4% in 2019), appears to have disappeared in recent years (0% in 2022 and 2023), possibly due to limited use.

Resistance to ciprofloxacin fluctuated over the years, with a peak of 28% in 2021, followed by a moderate decline. This variation may reflect shifting use patterns or the sporadic presence of resistant clones.

Amikacin resistance was present in the earlier years (6.5% in 2019, 8% in 2021) but dropped to 0% in 2022 and 2023, suggesting regained effectiveness and potential suitability as part of therapy for resistant *Citrobacter* infections (Table 5).

During the analyzed period, colistin resistance was also recorded in non-fermenting Gram-negative bacilli strains. In this study, a particular feature of colistin resistance within the hospital was that resistance was initially detected in *Enterobacterales* strains, followed by its emergence in non-fermenting Gram-negative bacilli.

### 2.6. Pseudomonas *spp.*

**Figure 8:**
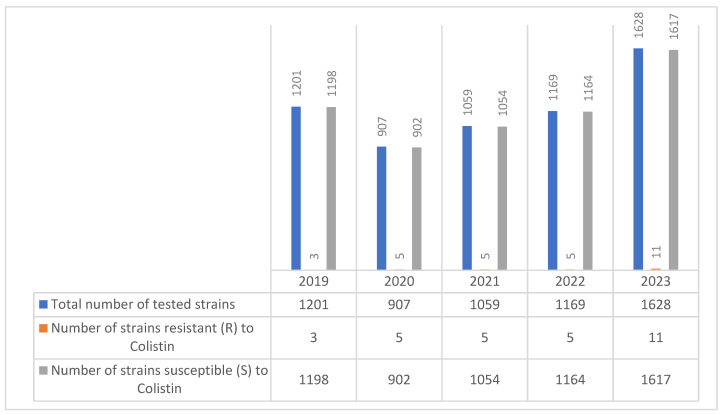
Evolution of colistin resistance in *Pseudomonas* spp. strains isolated during the period 2019–2023.

*Pseudomonas aeruginosa* colistin-resistant strains were first identified in our hospital in 2019 in small numbers, with a stable trend throughout the study period, peaking at 11 strains (0.67%) in 2023 (Figure 8).

A notable finding was that all strains were susceptible to ceftazidime-avibactam. Among the aminoglycosides, amikacin and tobramycin showed moderate efficacy, with susceptibility rates ranging between 48 and 53%. Resistance rates in *Pseudomonas* spp. were also recorded for cephalosporins and beta-lactamase inhibitors.

The resistance profile of *Pseudomonas* spp. over a five-year period highlights several clinically significant trends (Table 6).

Fluoroquinolones, particularly ciprofloxacin and levofloxacin, exhibited persistently high resistance rates, starting at approximately 60% in 2019 and gradually decreasing to approximately 41.5% and 38%, respectively, by 2023. Although a slight decrease was observed, resistance rates remain considerably high, indicating a reduced effectiveness of this antibiotic class for empirical therapy. Resistance to β-lactams, including piperacillin-tazobactam, ceftazidime, and cefepime, showed a notable decline across the study period. For example:

- Piperacillin-tazobactam resistance decreased from 42% in 2019 to 30% in 2023.
- Ceftazidime dropped from 53.5% to 29%.
- Cefepime fell from 53.8% to 20%. This trend may reflect changes in local prescribing practices or the displacement of resistant clones by more susceptible strains.

Carbapenem resistance (imipenem and meropenem) remained worryingly high, with initial rates exceeding 48% and decreasing only modestly to 38% and 30.8%, respectively, by 2023. These figures suggest persistent carbapenemase activity and ongoing transmission of carbapenem-resistant *Pseudomonas* strains.

Amikacin showed variable resistance, notably dropping to just 5% in 2020 but rising again in later years, suggesting episodic use or selective pressure shifts.

Ceftazidime-avibactam resistance decreased significantly from 30.9% in 2019 to 12.9% in 2023, supporting its role as a useful option for MDR *Pseudomonas*, especially given its activity against β-lactamase-producing strains.

Colistin exhibited very low resistance rates, remaining below 1% throughout the study period, underscoring its role as a last-resort therapeutic option. Nevertheless, even low levels of resistance call for caution given the limited treatment alternatives for MDR infections.

Several antibiotics tested against *Pseudomonas* spp. showed statistically significant changes in resistance rates over the 2019–2023 period, with *p*-values below 0.05 observed for piperacillin–tazobactam, ceftazidime, cefepime, ciprofloxacin, levofloxacin, and meropenem. These findings suggest a dynamic evolution of antimicrobial resistance in this species.

We identified several MDR *Pseudomonas* spp. phenotypes that circulated endemically, having been isolated from multiple patients (Table 7).

### 2.7. Acinetobacter *spp.*

**Figure 9:**
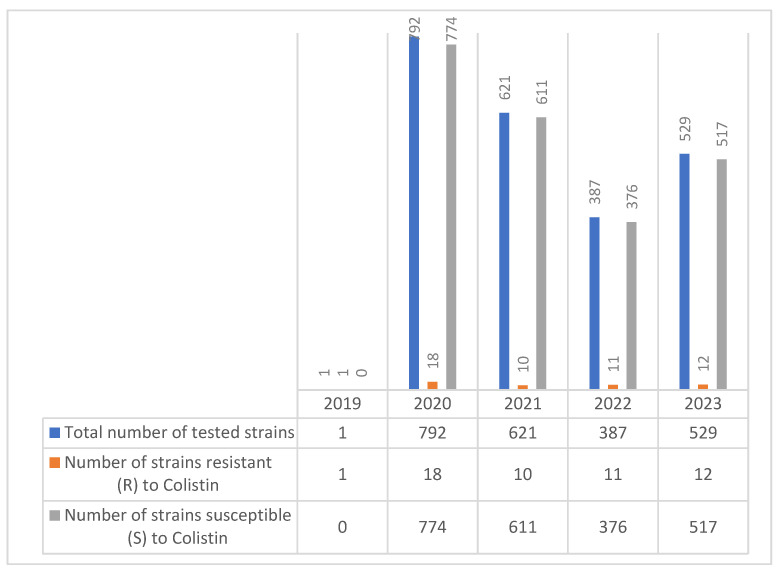
Colistin resistance in *Acinetobacter* spp. strains during the 2019–2023 period.

During the period 2019–2023, 2330 MDR *Acinetobacter* spp. were isolated, with 52 (5.43%) showing resistance to colistin (Figure 9).

The correlation analysis between year and resistance rates in *Acinetobacter* spp. isolates did not reveal any statistically significant temporal trends for any of the antibiotics tested (Table 8). Based on the data analyzed, *Acinetobacter* spp. strains showed marked resistance to meropenem, fluoroquinolones, and cephalosporins, while lower resistance rates were observed for colistin.

Colistin has proven effective in the treatment of infections caused by MDR *A. baumannii* strains, with over 97% of *Acinetobacter* spp. strains remaining susceptible to colistin in each of the five years studied (Table 8).

A variety of resistance phenotypes were observed among *Acinetobacter* spp. isolates, ranging from strains susceptible only to colistin to others retaining susceptibility to multiple agents such as tobramycin, amikacin, and trimethoprim-sulfamethoxazole (Table 9).

## 3. Discussion

The emergence and progression of antimicrobial resistance among Gram-negative bacilli, particularly colistin resistance, present a growing challenge in the clinical management of healthcare-associated infections. This five-year study offers a comprehensive overview of resistance trends in six key bacterial species: *Klebsiella* spp., *Escherichia coli*, *Enterobacter* spp., *Citrobacter* spp., *Pseudomonas* spp., and *Acinetobacter* spp., isolated from hospitalized patients in a tertiary care setting. *Klebsiella* spp. demonstrated the highest rate of colistin resistance among all tested isolates, representing more than 80% of the total colistin-resistant strains. This observation is consistent with global data, which highlight *Klebsiella pneumoniae* as a key contributor to colistin resistance, frequently associated with multidrug-resistant or extensively drug-resistant profiles. The high rates of resistance to carbapenems, cephalosporins, and fluoroquinolones further underscore the therapeutic difficulties posed by these isolates.

Our findings indicate that colistin resistance among *Klebsiella* spp. isolates in our hospital ranged between approximately 13% and 21.6% during the 2019–2023 period, with the highest rate recorded in 2020 (21.64%). These values significantly exceed those observed in earlier epidemiological studies. For instance, in Tunisia, resistance increased from 0.4% to 1.9% between 2005 and 2009 [33], while in Europe, resistance rates rose from 1.1% to 2.2% over the same period [33]. More recent surveillance data have shown elevated rates in specific countries, with Romania reporting up to 25.8%, followed by Greece (19.9%) and Italy (15.4%) [34]. Although the years of analysis differ, the comparison underscores a concerning pattern of colistin resistance emergence and persistence, particularly in regions with high antibiotic selection pressure.

Furthermore, more recent reports have documented a significant escalation in colistin resistance among carbapenem-resistant *K. pneumoniae*, with rates ranging between 27% and 71% in various regions across Asia, South Africa, and South America [35].

In contrast, *Escherichia coli* strains showed notably lower rates of colistin resistance, with most resistant strains exhibiting susceptibility to other antibiotic classes. This susceptibility profile suggests that plasmid-mediated resistance mechanisms, such as those associated with *mcr* genes, may be involved, rather than chromosomal mutations typically linked with broader resistance. Although rare, the presence of such strains warrants ongoing monitoring due to their potential for horizontal gene transfer and wider dissemination [36,37,38].

The predominance of colistin resistance among *K. pneumoniae* isolates in our hospital, compared to *E. coli*, aligns with findings from studies conducted in other countries, including China, Iran, and India [39,40,41].

*Enterobacter* spp. and *Citrobacter* spp. displayed low and relatively stable rates of colistin resistance throughout our study period. The absence of colistin resistance in *Citrobacter* spp. from 2021 onward is encouraging and may reflect limited selective pressure or effective antimicrobial stewardship.

The results of our study indicate that, over the five-year period (2019–2023), colistin resistance among *Enterobacter* spp. remained relatively stable, with annual resistance counts ranging from 9 to 13 out of a total of 1116 tested strains—corresponding to yearly resistance rates of approximately 3% to 5%. This persistent yet moderate level of resistance did not show a clear upward or downward trend over time. These findings are in line with those reported by Mirelis et al., who found that while the overall colistin resistance rate among *Enterobacteriaceae* was 0.67%, *Enterobacter cloacae* exhibited a considerably higher resistance rate of 4.2%, exceeding those of *Escherichia coli* and *Klebsiella pneumoniae* [42].

Throughout the five-year observation period, our findings revealed that colistin resistance among *Citrobacter* spp. remained exceptionally low, with only isolated cases identified in 2019 and 2020 (2–2.7%), and no resistant strains detected in the subsequent years (2021–2023). This trend suggests stable susceptibility and limited selective pressure associated with colistin use in our clinical setting. In contrast, a recent study by Puljko et al., conducted in 2024, reported a higher resistance rate of 5% among *Citrobacter* isolates, indicating possible regional or institutional differences in antimicrobial usage patterns or resistance dynamics [43].

*Pseudomonas* spp. and *Acinetobacter* spp. remain prominent nosocomial pathogens due to their intrinsic resistance mechanisms and ability to acquire additional resistance determinants [44].

Our data show consistently low colistin resistance in *Pseudomonas* spp. isolates, remaining below 1% throughout 2019–2023. This finding reinforces the continued efficacy of colistin as a last-line therapeutic agent in our institution. However, even minimal resistance remains a cause for concern due to the limited treatment alternatives available for multidrug-resistant infections. In contrast, international data reveal a more variable resistance landscape. For instance, a recent study in Ardabil, Iran, reported a colistin resistance rate of 9% in *P. aeruginosa*, marking the first such report from that region [45]. Other studies across Iran and globally have documented resistance rates ranging from 0% to over 21%—including 21.3% in Egypt, 6.3% in Brazil, 7.4% in South Korea, and 7.2% in Peru [46,47,48,49]. Lower resistance rates were reported in regions such as Shiraz, Iran (0%), and Tehran, Iran (0%), whereas other Iranian cities like Isfahan and Hamadan reported rates of 3.9% to 7% [50,51,52,53,54,55]. These discrepancies underscore the influence of local antimicrobial stewardship practices, infection control measures, and selective pressure on colistin resistance dynamics.

Although colistin resistance remained low, below 1% for *Pseudomonas* spp. and around 2–3% for *Acinetobacter* spp., the high resistance rates to carbapenems and fluoroquinolones observed in our study are concerning and underscore the ongoing challenges in managing infections caused by multidrug-resistant Gram-negative bacilli. Notably, *Pseudomonas* spp. showed a gradual improvement in susceptibility to β-lactams, possibly reflecting shifts in antimicrobial usage or infection control successes. Meanwhile, *Acinetobacter* spp. exhibited persistently high resistance to nearly all tested antibiotics, reinforcing the clinical reliance on colistin as a last-resort agent.

Among the *Acinetobacter* spp. isolates analyzed in our research, only 52 out of 2330 strains were resistant to colistin, reflecting a relatively low overall resistance rate of 5.43%. Annual resistance rates fluctuated between 0% and 2.84%, suggesting limited but persistent occurrence of colistin resistance within this group of multidrug-resistant pathogens. Notably, over 97% of *Acinetobacter* spp. strains remained susceptible to colistin in each of the five years analyzed, underscoring its continued clinical utility in our setting.

These findings are in line with earlier data from Mohammadi et al. [56], who reported a 2% resistance rate in a 2017 meta-analysis of 448 *A. baumannii* strains. However, more recent studies have demonstrated increasing resistance rates globally. A meta-analysis by Ciftci et al. in Turkey revealed a rise in colistin resistance from 2.94% (2011–2015) to 13.42% (2016–2020), with an overall resistance rate of 7.9% [57]. Additionally, individual reports from India, Pakistan, Turkey, and Sweden have shown even higher colistin resistance rates of 10.1%, 9.6%, 28%, and 36.36%, respectively [58,59,60,61].

In addition to colistin resistance, our data revealed complex and diverse MDR phenotypes among *Klebsiella* spp., *Pseudomonas* spp., and *Acinetobacter* spp. isolates. The presence of strains resistant to nearly all tested antibiotic classes, including carbapenems, aminoglycosides, and fluoroquinolones, underscores the growing challenge of treating infections caused by these pathogens. Particularly concerning were *Klebsiella* spp. phenotypes that remained susceptible only to last-resort agents such as colistin. These patterns are consistent with known resistance mechanisms such as carbapenemase production (e.g., KPC, OXA-48), efflux pump overexpression, and porin loss [62].

The clinical implications are significant, as treatment options become increasingly limited, often requiring combination therapies or off-label use of less validated agents. These findings align with international reports and highlight the importance of local surveillance data to guide empirical therapy and stewardship efforts.

Overall, our study reveals that while colistin resistance remains relatively low in most Gram-negative species (except *Klebsiella* spp.), the threat of emerging resistance persists.

## 4. Materials and Methods

### 4.1. Isolation and Identification

A retrospective-prospective study was conducted over a five-year period (2019–2023) at “Sf. Spiridon” County Clinical Emergency Hospital in Iași, Romania. During this time, a total of 23,143 Gram-negative bacilli (GNB) strains were isolated, of which 14,849 were classified as *Enterobacterales* and 8294 as non-fermenting GNB. Out of the total GNB isolates, 4795 strains were identified as MDR, and 910 were colistin-resistant. All isolates were identified using matrix-assisted laser desorption ionization–time-of-flight mass spectrometry (MALDI-TOF MS) (Bruker Daltonik GmbH, Bremen, Germany) or conventional biochemical assays.

All clinical specimens analyzed in this study were obtained from hospitalized patients as part of standard diagnostic and therapeutic protocols at “Sf. Spiridon” County Clinical Emergency Hospital, Iași, Romania. No additional sampling was performed for research purposes. The analysis was conducted exclusively on samples routinely submitted to the microbiology laboratory, which included wound cultures, endotracheal aspirates, urine, blood, serous fluids, abscess contents, and other pathological specimens. These were collected from a broad range of hospital departments, including internal medicine wards, surgical units, and intensive care departments.

### 4.2. Antimicrobial Susceptibility

Antimicrobial susceptibility testing was performed using the broth microdilution method, in accordance with the EUCAST guidelines applicable at the time of testing (versions 9.0–13.0, 2019–2023), using an automated system (MICRONAUT-S, Merlin, Bornheim, Germany). For colistin, susceptibility testing was specifically conducted using the standard broth microdilution method, the only method recommended by EUCAST and CLSI for reliable results. Minimum inhibitory concentrations (MICs) were interpreted according to EUCAST clinical breakpoints. For quality control, standard reference strains such as *Escherichia coli* ATCC 25922 and *Pseudomonas aeruginosa* ATCC 27853 were included in each testing series [63,64,65,66,67]. Antibiogram results were analyzed using the Statistical Package for the Social Sciences (SPSS^®^ Statistics, version 25; IBM Corp., Armonk, NY, USA). Associations between the year of isolation and resistance rates for each antibiotic were evaluated using the Pearson Chi-square test. A *p*-value of <0.05 was considered indicative of statistical significance.

For colistin, both the Clinical and Laboratory Standards Institute (CLSI) and EUCAST recognize broth microdilution, performed in accordance with ISO 20776 [68], as the gold standard method for susceptibility testing [69].

In our study, the definition of multidrug resistance (MDR) follows the criteria proposed by Magiorakos et al. in collaboration with the European Centre for Disease Prevention and Control (ECDC) and the Centers for Disease Control and Prevention (CDC). An isolate was considered MDR if it showed acquired non-susceptibility to at least one agent in three or more antimicrobial categories relevant to that species [70].

## 5. Conclusions

This study highlights the emergence and progressive increase in colistin resistance, as well as resistance to commonly used antibiotics among Gram-negative bacilli strains. Within the framework of this study, we provide a comprehensive overview of antibiotic resistance in the North-East region of Romania.

The analysis of the collected data allowed us to evaluate the current profile of colistin resistance among clinical isolates of Gram-negative bacilli.

Our research emphasizes the importance of assessing the local epidemiological context and the need for continuous monitoring of antibiotic resistance, with the aim of ensuring rapid diagnosis and appropriate antimicrobial prescription.

Moreover, the study reveals a diverse range of multidrug-resistant (MDR) phenotypes circulating in our region, particularly among *Klebsiella* spp., *Pseudomonas* spp., and *Acinetobacter* spp., underscoring the therapeutic challenges posed by these pathogens.

## Figures and Tables

**Table 1:** Evolution of antibiotic resistance (%) in *Klebsiella* spp. strains between 2019 and 2023.

Antibiotic	Year					
	2019	2020	2021	2022	2023	*p*-Value
Amikacin	5.63%	12.86%	5.63%	15.61%	23.38%	0.096
Cefotaxime	52.85%	57.75%	55.06%	55.08%	55.09%	0.791
Ceftazidime	50.89%	53.98%	50.38%	52.7%	52.55%	0.721
Ceftriaxone	50.55%	58.09%	53.44%	53.64%	54.18%	0.791
Cefepime	47.09%	44.96%	46.43%	48.99%	48.97%	0.176
Ciprofloxacin	42.76%	49.19%	49.37%	49.8%	48.71%	0.213
Colistin	12.99%	21.64%	12.97%	14.06%	15.82%	0.894
Imipenem	22.55%	19.20%	31.34%	32.48%	31.68%	0.094
Meropenem	25.37%	29.39%	30.88%	32.46%	31.82%	0.041
Ertapenem	25.64%	34.35%	34.35%	37.79%	36.95%	0.065
Trimethoprim-sulfamethoxazole	42.14%	49.01%	47.89%	48.73%	50.58%	0.097

**Table 2:** *Klebsiella* spp. resistance phenotypes.

Phenotype	Antibiotics				
	IMI	MEM	ETP	CN	AK
Phenotype 1	S	I			
Phenotype 2	I			S	
Phenotype 3				S	S
Phenotype 4		S	S		S
Phenotype 5					S

Note: IMI = Imipenem, MEM = Meropenem, ETP = Ertapenem, AK = Amikacin, CN = Gentamicin; S = Susceptible, I = Intermediate; Phenotype 1 = Susceptible only to IMI and Intermediate to MEM; Phenotype 2 = Susceptible only to CN and Intermediate to IMI; Phenotype 3 = Susceptible only to CN and AK; Phenotype 4 = Susceptible only to MEM, ETP, and AK; Phenotype 5 = Susceptible only to AK. Green shading indicates antibiotic susceptibility; yellow shading indicates intermediate susceptibility.

**Table 3:** Evolution of antibiotic resistance in *Escherichia coli* strains between 2019 and 2023.

Antibiotic	Year					
	2019	2020	2021	2022	2023	*p* Values
Amoxicilin & clavulanic acid	32.26%	39.59%	37.27%	24.88%	29.18%	0.314
Amikacin	0.68%	1.64%	0.82%	0.60%	0.71%	0.548
Cefotaxime	26.01%	28.04%	18.78%	16.52%	16.53%	0.048
Ceftazidime	20.40%	22.41%	14.58%	12.26%	12.63%	0.054
Ceftriaxone	33.33%	26.64%	18.64%	16.48%	16.75%	0.024
Cefepime	19.01%	17.51%	12.94%	10.69%	12.49%	0.046
Ciprofloxacin	26.75%	30.33%	29.22%	22.68%	21.70%	0.161
Colistin	1.25%	0.94%	0.18%	0.31%	0.48%	0.133
Imipenem	0.52%	1.70%	0.95%	1.26%	2.89%	0.144
Meropenem	0.67%	1.63%	1.19%	0.05%	0.04%	0.244
Ertapenem	0.92%	2.46%	2.01%	1.40%	1.069%	0.765
Trimetoprim & sulfametoxazol	29.38%	36.30%	32.85%	25.38%	30.49%	0.577
Nitrofurantoin	3.27%	2.05%	3.45%	2.78%	3.75%	0.503

**Table 4:** Evolution of antibiotic resistance in *Enterobacter* spp. strains between 2019 and 2023.

Antibiotic	Year					*p*-Values
	2019	2020	2021	2022	2023	
Amikacin	4.96%	4.78%	4.65%	2.25%	5.88%	0.897
Cefotaxime	37.5%	42.70%	38.15%	32.81%	32.41%	0.147
Ceftazidime	33.66%	39.79%	32.18%	32.95%	31.52%	0.359
Ceftriaxone	35.11%	42.78%	34.51%	31.43%	30.50%	0.214
Cefepime	18.50%	21.26%	20%	16.34%	17.58%	0.337
Ciprofloxacin	19.70%	28.79%	27.04%	19.04%	23.95%	0.942
Colistin	3.97%	5%	5.77%	3.58%	5.46%	0.672
Imipenem	2.55%	5.23%	5.98%	4.15%	8.15%	0.131
Meropenem	4.14%	5.26%	6.83%	3%	5.29%	0.994
Ertapenem	5.28%	7.10%	7.58%	9.26%	12.92%	0.011

**Table 5:** Evolution of antibiotic resistance in *Citrobacter* spp. strains between 2019 and 2023.

Antibiotic	Year					*p*-Values
	2019	2020	2021	2022	2023	
Amikacin	6.52	2.94	8	0	0	0.074
Cefotaxime	27.16	27.77	39.13	42.85	36.36	0.027
Ceftazidime	24.77	24.32	36	37.5	36.36	0.003
Ceftriaxone	25.45	27.02	32	37.5	50	0.146
Cefepime	4	2.94	16.66	0	0	0.557
Ciprofloxacin	14.91	7.69	28	12.5	18.18	0.702
Colistin	2.04	2.77	0	0	0	0.099
Imipenem	2.80	2.70	0	0	0	0.055
Meropenem	1.86	0	4	0	0	0.586
Ertapenem	2.83	2.70	8	0	0	0.499

**Table 6:** Evolution of antibiotic resistance in *Pseudomonas* spp. strains between 2019 and 2023.

Antibiotic	Year					*p*-Values
	2019	2020	2021	2022	2023	
Piperacilin & tazobactam	42.02%	42.99%	41.37%	33.77%	30.34%	0.033
Amikacin	17.27%	5.09%	19.90%	26.17%	20.49%	0.329
Ceftazidime	53.51%	45.83%	48.55%	33.22%	28.99%	0.02
Cefepime	53.81%	42.37%	40.81%	22.50%	20.06%	0.007
Ceftazidime & avibactam	30.86%	35.06%	31.68%	14.17%	12.89%	0.066
Ciprofloxacin	59.72%	59.09%	55.89%	45.34%	41.53%	0.013
Levofloxacin	60.48%	60.06%	56.06%	42.46%	37.99%	0.016
Imipenem	48.20%	43.04%	48.80%	40.54%	38.05%	0.131
Meropenem	53.83%	45.09%	46.10%	35.12%	30.77%	0.009
Colistin	0.24%	0.55%	0.47%	0.42%	0.67%	0.167

**Table 7:** *Pseudomonas* spp. resistance phenotypes.

Phenotype	Antibiotics						
	CT	TZP	TOB	AK	CIP	LEV	CAZ
Phenotype 1	S						
Phenotype 2	S	I					
Phenotype 3	S	S	S	S			
Phenotype 4	S	S			S	S	S
Phenotype 5	S	S					S

Note: CT = Colistin, TZP = piperacillin&tazobactam, TOB = Tobramycin, AK = Amikacin, CIP = Ciprofloxacin, LEV = Levofloxacin, CAZ = Ceftazidime; S = Susceptible; I = Intermediate. Phenotype 1 = Susceptible only to CT; Phenotype 2 = Susceptible to CT and Intermediate to TZP; Phenotype 3 = Susceptible to CT, TZP, TOB, and AK; Phenotype 4 = Susceptible to CT, TZP, CIP, LEV, and CAZ; Phenotype 5 = Susceptible to CT, TZP, and CAZ. Green shading indicates antibiotic susceptibility; yellow shading indicates intermediate susceptibility.

**Table 8:** Evolution of antibiotic resistance in *Acinetobacter* spp. strains between 2019 and 2023 period.

Antibiotic	Year					*p*-Values
	2019	2020	2021	2022	2023	
Amikacin	5.63%	73.96%	83.06%	85.34%	90.60%	0.094
Gentamicin	90.87%	92.58%	82.32%	80.58%	91.47%	0.621
Ciprofloxacin	97.31%	96.05%	94.10%	93.02%	95.48%	0.255
Levofloxacin	95.90%	95.46%	94.10%	93.02%	95.49%	0.471
Imipenem	91.02%	94.06%	92.93%	90.23%	94.56%	0.657
Meropenem	94.64%	94.17%	91.82%	90.23%	94.37%	0.545
Colistin	0	2.27%	1.61%	2.84%	2.26%	0.157

**Table 9:** *Acinetobacter* spp. resistance phenotypes.

Phenotype	Antibiotics			
	CT	TOB	AK	SXT
Phenotype 1	S			
Phenotype 2	S	S		
Phenotype 3	S	S		S
Phenotype 4	S	S	S	
Phenotype 5	S			S
Phenotype 6	S	S	S	S

Note: CT = Colistin, TOB = Tobramycin, AK = Amikacin, SXT = Trimethoprim–sulfamethoxazole. S = Susceptible (green); Phenotype 1 = Susceptible only to CT; Phenotype 2 = Suscepetible to CT and TOB; Phenotype 3 = Susceptible to CT, TOB, and SXT; Phenotype 4 = Susceptible to CT, TOB, and AK; Phenotype 5 = Susceptible to CT and SXT; Phenotype 6 = Susceptible to CT, TOB, AK, and SXT. Green shading indicates antibiotic susceptibility.

## Data Availability

The original contributions presented in this study are included in the article. Further inquiries can be directed to the corresponding author.
